# Intraspecific Relationships and Nest Mound Shape Are Affected by Habitat Features in Introduced Populations of the Red Wood Ant *Formica paralugubris*

**DOI:** 10.3390/insects13020198

**Published:** 2022-02-14

**Authors:** Filippo Frizzi, Alberto Masoni, Margherita Santedicola, Martina Servini, Nicola Simoncini, Jessica Palmieri, Giacomo Santini

**Affiliations:** Department of Biology, University of Florence, Via Madonna del Piano 6, 50019 Florence, Italy; filippo.frizzi@unifi.it (F.F.); alberto.masoni@unifi.it (A.M.); margheritasantedicola@yahoo.it (M.S.); martina.servini@gmail.com (M.S.); nico1111993@hotmail.it (N.S.); jess.palmieri07@gmail.com (J.P.)

**Keywords:** red wood ants, introduced populations, nest mound shape, soil material, unicoloniality, trail networks

## Abstract

**Simple Summary:**

Red wood ants (*Formica rufa* goup) are dominant ant species widespread in the Eurasian continent. These species have a strong ecological impact on the habitats they dwell in, being top-ranked predators. One of the most striking features of these ants is represented by the large nest mounds they build. In this study, we investigated how nest mound shape and colony organization of imported populations of *Formica paralugubris* varied in three different habitat types. We found that nest mounds differed in size, number and shape in the three habitats. In all the three sites, nests were connected by trails of workers, but the size of these nest-networks differed. We also investigated the pattern of intraspecific aggression among ants from different nests, and we showed that aggressiveness was higher within each population than between separate populations, a finding in line with a ‘nasty neighbor’ behavior.

**Abstract:**

Ants belonging to the *Formica rufa* group build large nest mounds, which aid their survival during severe winters. We investigated whether different environmental features of the habitats affected the nest mound shape and the population structure. We assessed the shape of all the nest mounds and mapped inter-nest trails connecting mounds for three imported populations of *Formica paralugubris* in three forest habitats: fir-dominated, beech-dominated, and a mixture of fir and beech. Single-nest mounds were averagely smaller and flatter in the beech-dominated forest, probably because of lighter building materials. Nonetheless, by summing the volumes of all interconnected nests, the size was similar among all three sites. In fir- and beech-dominated forests, large nests were also central in the networks, suggesting a central place foraging model with these nests as reference. We finally performed aggression tests, and found that aggressiveness was significantly higher among nests belonging to the same population than between populations. The results highlight the plasticity of the species to adapt nest and colony structure to different environments. Additionally, it appears that none of these populations is unicolonial, as observed in various alpine sites, there and the observed patterns of aggression are coherent with the ‘nasty neighbor’ effect.

## 1. Introduction

Ants belonging to the *Formica rufa* group, commonly known as red wood ants (RWAs), are widespread throughout the mountain and continental forests of Europe [1]. One of the most striking features shared by these cold-adapted species is the construction of large epigeous nest mounds that protect their inner chambers from severe winter conditions [2] and modify the chemical characteristics and nutrient concentration of the soil [3,4,5,6]. The materials used by workers to build such structures are highly variable, and include soil particles, small pebbles, resin granules, twigs, and other litter elements [7]. One of the most important components of the mounds are the needle-shaped leaves of coniferous trees [8], although it is still unclear how much this choice is influenced by their availability in the habitats they colonize [9].

Several RWA species are polydomous, i.e., the colonies are subdivided into spatially separated but interconnected nests, each housing a portion of the workforce and brood [10]. Nest fission or budding is a phenomenon where new nests are founded near the mother colony and are initially occupied by a small group of workers with one or a few queens [11]. This biological trait may lead to the onset of polydomy, as a relationship between the new nests and their mother colony is maintained. Polydomy in some RWA species can be flexible according to habitat type [12] and resource distribution [13], and in some contexts monodomy can also occur [14]. The identification and analysis of trail networks connecting different nests in polydomous species can provide important insights on foraging efficiency and the subdivision of tasks within a colony [15,16].

*Formica paralugubris* was described in the mid-nineties and native populations are restricted to the European Alps [17]. Alpine ant populations studied to date exist as supercolonies, with behavioral relationships among the nests suggesting unicoloniality [18,19]. Unicolonial supercolonies are polydomous colonies composed of many nests across vast areas, where workers are only weakly related and are free to move between nests without eliciting aggression from non-nestmates [20]. Over several decades, hundreds of nests of this alpine species were collected and introduced outside of their natural range of distribution as biological control agents against arthropod pests [21,22]. Some of these ant populations introduced into the Apennine forests in Italy have survived and are now expanding [23]. In some sites, such as the Campigna Biogenetic Nature Reserve (Central Italy), populations were introduced at locations spatially segregated by several hundred meters or a few kilometers [24,25]. The majority of introductions occurred in fir-dominated forests (*Abies alba*), which resemble the original alpine habitat [26], but also in forests with increasing beech (*Fagus sylvatica*) density. It is known that environmental context and isolation are two factors that may influence colony arrangement and intraspecific relationships of RWA populations [12,27], and we may therefore expect that these populations may differ to some extent. Recently, it has been demonstrated that RWAs prefer conifers to forage on aphids, a detail suggesting a harder adaptation to broadleaves forests [28]. Assessing the relationships among nests in populations located in different habitats may provide interesting insights on the ecological plasticity of this species, which is, to date, only known to be unicolonial. Furthermore, it is known that RWAs adapt their nest-building according to available materials [21]; thus, we expected to find different mound structures in the different populations. 

In this study, we selected three geographically close but distinct populations, characterized by different tree composition (fir-dominated, mixed fir-beech, beech-dominated), and whose colonial arrangement was unknown. In the three selected populations we compared nest mound size and shape, and we assessed the type of nest material used. To check if differences in mound size and form were due to structural properties of the material used, we experimentally calculated the angle of repose of each class of materials [29]. Finally, we mapped connecting trails between nests to identify colony boundaries, and to check whether these populations were unicolonial as those in the Alps, we performed aggression tests between workers from different colonies, both within and between populations.

## 2. Materials and Methods

**Figure 1:**
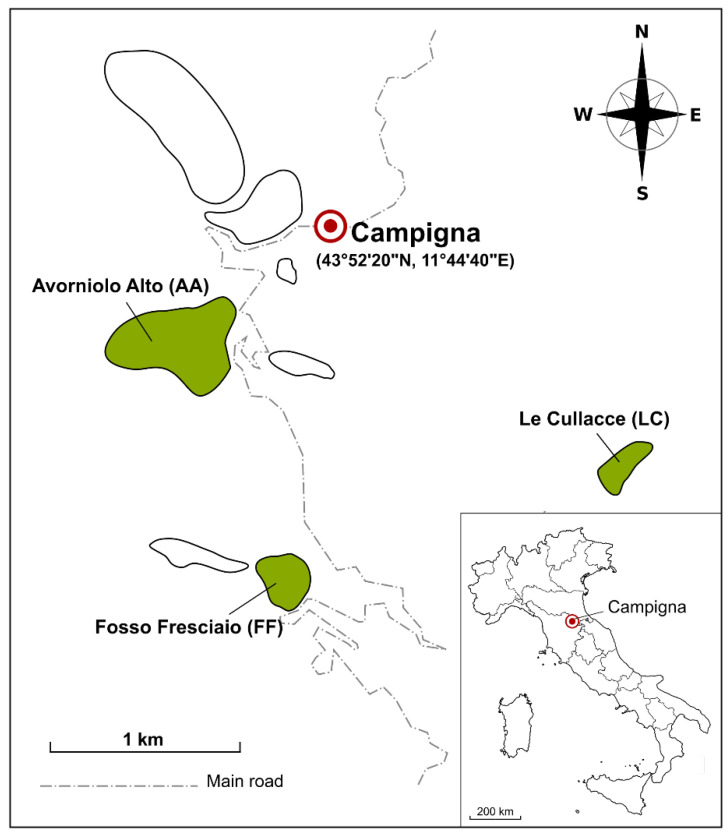
Map of *F. paralugubris* populations in the Campigna Biogenetic Nature Reserve. Green areas indicate the three populations analyzed in this study and white areas are populations not analyzed in this study. AA, fir–dominated forest; LC, mixed forest; FF, beech–dominated forest.

A census of the nests of three *F. paralugubris* populations introduced into the Campigna Biogenetic Nature Reserve, Italy (43°52′20″ N, 11°44′40″ E) was performed from June to July 2016. The specific sites, which will be used to refer to the populations, were Avorniolo Alto (AA), a fir-dominated forest with sparse beeches; Le Cullacce (LC), a mixed forest with beeches and firs in approximately equal proportions; and Fosso Fresciaio (FF), a beech-dominated forest with sparse firs (Figure 1). The GPS coordinates and measurements of total and half-height, upstream to downstream base width, and the width perpendicular to this last measure were recorded for each nest.

**Figure 2:**
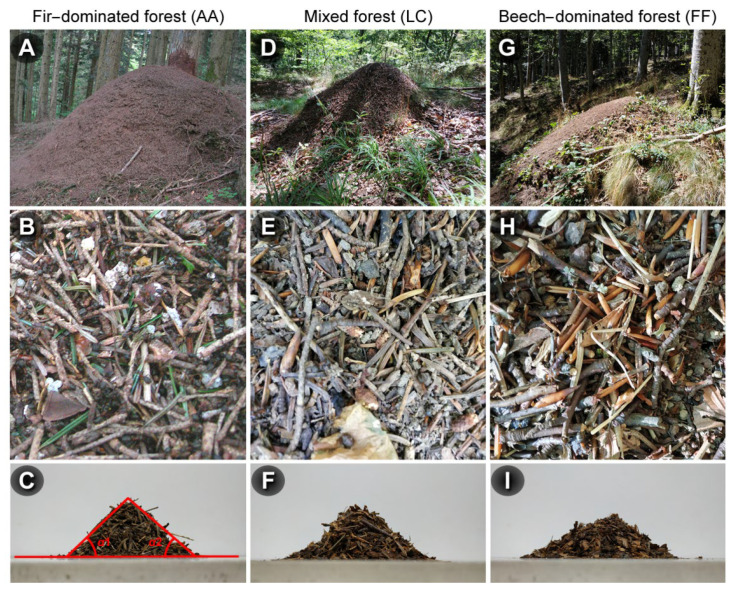
Examples of mound shape (**A**,**D**,**G**), nesting material composition (**B**,**E**,**H**), and angle of repose (**C**,**F**,**I**) at the three sites. In C, the two angles (α1 and α2) used for assessing the angle of repose are indicated in red, with the average of these values reported.

The volume of each nest was calculated using the method described by Ronchetti et al. [24] and used by Frizzi et al. [25]. Formulas for calculating volumes were:

$$V_{p}=\frac{\pi hl^{2}}{8}$$

and:

$$V_{h}=\frac{\pi h\left(l^{2}+4l_{1}^{2}\right)}{24}$$

where *h* is the total height, *l* is the average between the larger and the shorter diameter at the base, and *l*_1_ is the half-height of the mound. The first formula was applied when mound height was equal to or greater than 75% of the mean base diameter, the second otherwise. As a summary measure to describe nest shape, we used the ratio between the height and the mean width of the base (here called size–ratio index, SRI). The lower the value of this measure, the flatter the nest. We subdivided all the SRI values into three intervals according to the 25, 75 and 100 quantiles, and the difference in the frequency distribution among the three sites was assessed by the Pearson’s chi square test. To investigate the effect of the nest material on the shape of the nest mounds, we collected ≥0.5 L of nest material (15 cm deep) from 10 randomly chosen nests/site and we assessed the angle of repose, which can provide important information about the physical properties of the material [29]. Small nests (<150 L) were excluded from sampling. All samples were transported to the laboratory within 3 h of collection, where they were stored in 5-cm diameter, 15-cm tall glass cylinders. Cylinders were capped with a plastic sheet, overturned onto a smooth surface, and slowly lifted following removal of the plastic sheet. The angle of repose for each sample was calculated as the mean angle of the two slopes of the resulting heap, from the top to the base margins (Figure 2). We also performed an analysis of the materials composing ten randomly chosen nests from each site.

All the nests and their surrounding area were carefully examined to identify trails of workers connecting different nests. A nest network is defined as a group of nests connected by trails of workers [15]. As nest networks are highly dynamic [30], all nests in each site were evaluated three times at two-week intervals. All nests connected in at least one of these sampling events were considered as belonging to the same network. We focused on the relationships among nests, thus foraging trails towards trees were not considered. For each network, we computed the size (i.e., number of nests), total volume (i.e., the sum of the volumes of all component nests), and the betweenness centrality. The betweenness centrality is the number of shortest trails passing by each nest (i.e., a node in the network jargon) considering all possible connections between pairs of nests in the network [31]. This may be an indication whether colonies are organized with some central nests as a reference [13].

We performed group aggression tests between equally-sized ants from different nests belonging to different networks, from both the same and different populations [32]. Different networks are not necessarily equivalent to different colonies [33], but connected nests share individuals and cannot be considered as independent in dyadic aggression tests. Five ants from each nest were used in each test. We performed 10 tests within each population and 25 tests for each pair of populations, giving a total of 105 tests. We also performed 10 control tests for each site using ants collected from nests of the same network. No more than 15 tests were conducted each day, with all tests performed on sunny days. Twenty workers or fewer were collected with a small brush, to minimize manipulation stress, into a 50 mL Falcon tube, which was sealed with a fine mesh to allow air circulation. Ants were transported to a field laboratory within 15 min of collection and were acclimatized for 20 min prior to performing the aggression tests. Paired groups were simultaneously dropped into a neutral arena constructed from a 4.5 cm Petri dish with Fluon^®^-coated walls to prevent escape. Aggressive behaviors were scored as 0 or 1, with 0 indicating no aggression (avoidance, short antennation, trophallaxis) or attention (prolonged antennation, retreat following contact) behaviors and 1 indicating aggressive displays (open mandibles, gaster flexion) or physical aggression (biting, venom spraying). Ants were observed for 5 min, and the test was scored based on the most aggressive behavior detected. All tests were blind, with the ants’ provenance unknown to the observers [34].

All comparisons were carried out using generalized linear models (GLM). Gaussian models were fitted to analyze volume sizes and angles of repose, with the site as main factor. Poisson models with the Wald *χ*^2^ test were used to compare the number of nests forming a network within each site and the betweenness centrality; in the former, the site was the main factor, whereas in the second the betweenness was compared with the nest volume. Finally, logistic regressions were used to analyze aggression tests, and the type of test, i.e., intra-site or inter-site, was the main term [35]. Tukey’s test was used for multiple comparisons. Nest volumes were transformed by logarithm because of the great difference between them. All analyses were performed using R version 4.0.1 software [36].

## 3. Results

**Figure 3:**
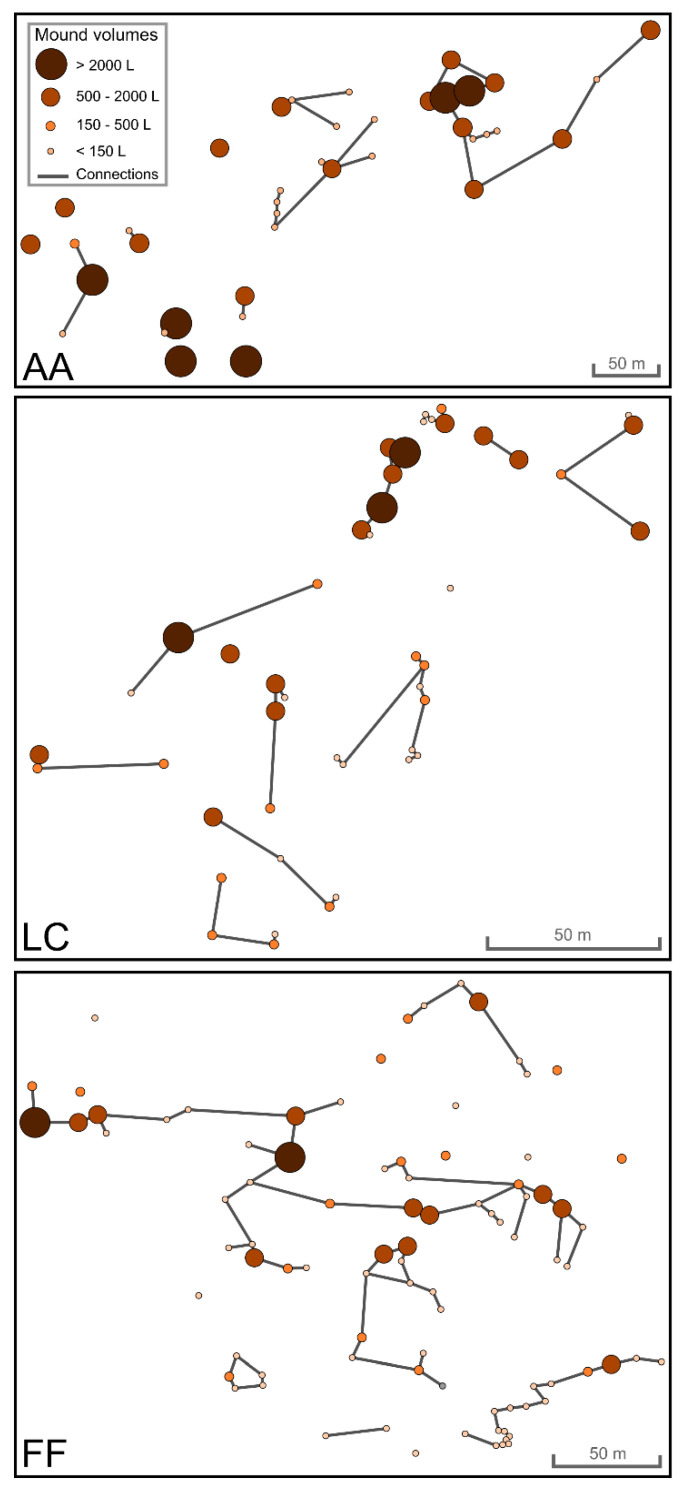
Map of nest network connections (lines) indicating nest size (circle markers) and location at the three sites. AA: Avorniolo Alto, fir–dominated forest; LC: Le Cullacce, mixed forest; and FF: Fosso Fresciaio, beech–dominated forest.

Thirty-nine nests were mapped at AA, 46 at LC, and 88 at FF (Figure 3). Examples of nest mound shape, composition, and angle of repose of the nesting material in the three sites are shown in Figure 2. 

**Figure 4:**
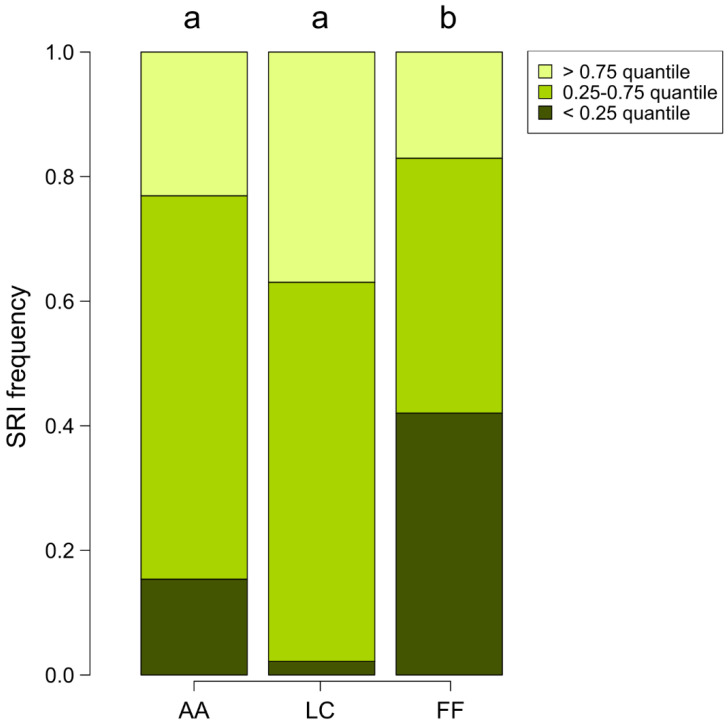
Barplot of the frequency distribution of the SRI index (height of the nest divided by the mean width of its base) in the three sites. AA: Avorniolo Alto, fir-dominated forest; LC: Le Cullacce, mixed forest; and FF: Fosso Fresciaio, beech-dominated forest. Letters above bars represent the significance of multiple comparison tests.

The frequency distribution of the SRI index differed among sites (*χ*^2^ = 29.44, df = 4, *p* < 0.001, (Figure 4). In particular, FF significantly differed from AA and LC, having a higher amount of low SRI values (i.e., flatter nests). On the contrary, no significant difference was found between AA and LC. These distributions were significantly different between AA and FF (*χ*^2^ = 8.63, df = 2, *p* = 0.013) and between LC and FF (*χ*^2^ = 24.47, df = 2, *p* < 0.001), but not between AA and LC (*χ*^2^ = 5.80, df = 2, *p* = 0.055). In these last tests, the significance must be considered with α < 0.017 after the Bonferroni correction for multiple comparisons. 

**Figure 5:**
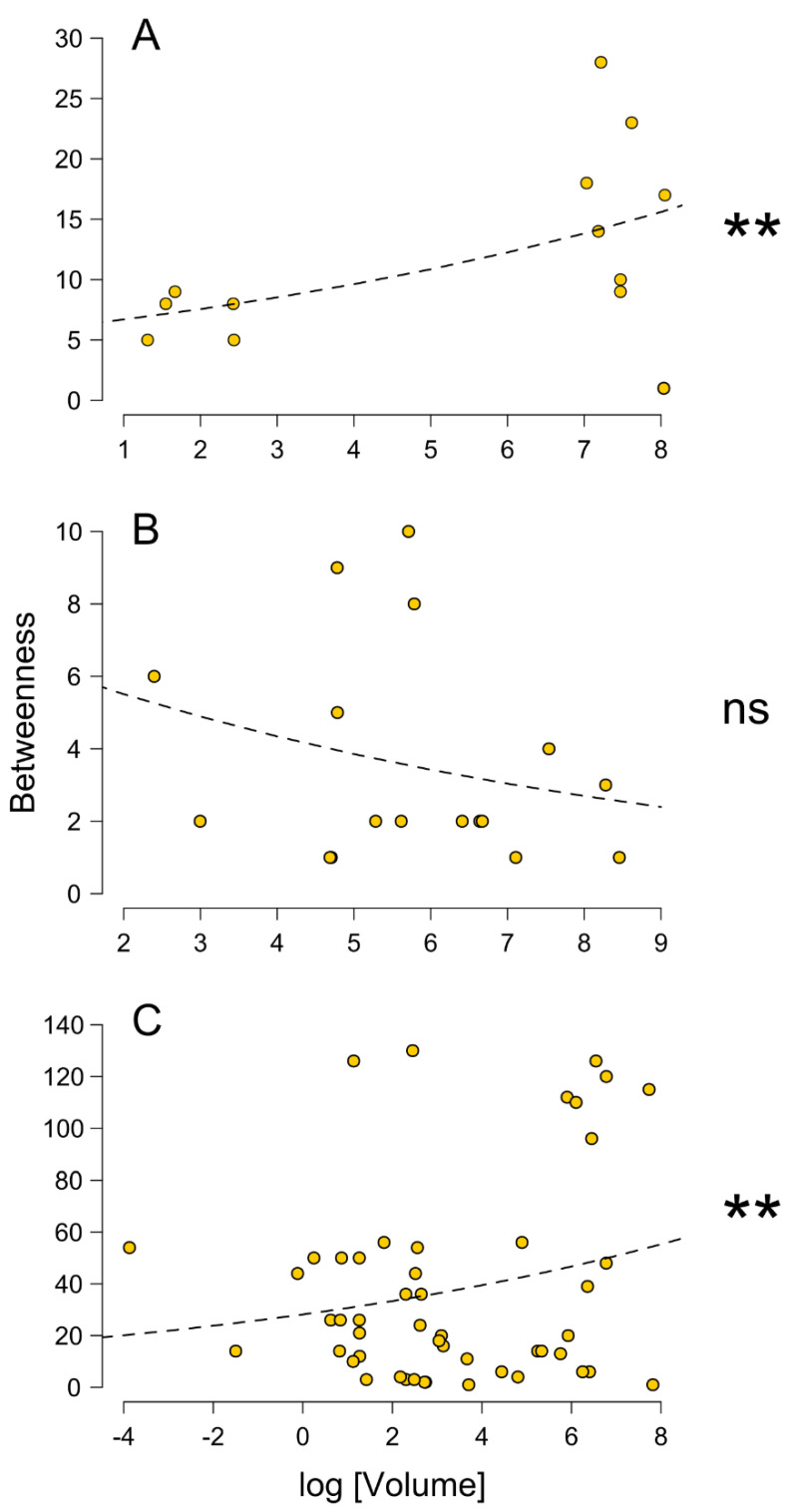
Relation between betweenness and volumes of nest mounds (transformed by logarithm) in the three sites, with models fitted (dashed lines). Each symbol is a nest having a betweenness >0. Asterisks represent the significance of the model (** *p* < 0.001). ns = Not significant. (**A**), Avorniolo Alto (AA), fir–dominated forest; (**B**), Le Cullacce (LC), mixed forest; (**C**), Fosso Fresciaio (FF), beech–dominated forest.

The nest materials were similar (e.g., small twigs and soil granules), although nests in FF and LC contained varying proportions of beech buds in place of fir needles. Considering the number of fir needles and beech buds comprised in the nest material observed, the latter represented approximately 63% (±5.31SE) of the total at FF and 13% (±2.16SE) at LC, while no beech buds were found in AA nests. While beech leaf fragments were observed in FF nests, they were rare or absent in AA and LC. The mean angle of repose of nest materials differed significantly between the sites (F_2,27_ = 13.4, *p* < 0.001), being narrower in FF than in the two other sites that did not differ between them. The average size of the networks differed across the sites (χ^2^ = 7.19, *p* = 0.027), increasing from LC (range 1–9) to AA (range 1–13) to FF (range 1–35). In a pairwise comparison, the only significant difference occurred between FF and AA (z value = 2.483, *p* = 0.034). Despite the difference in network size, the average network volumes were not significantly different (F_2,37_ = 0.764, *p* = 0.473). Additional details are reported in the Supplementary Table S1. Betweenness was positively related with the mound volume both in AA (*χ*^2^ = 14.86, *p* < 0.001) and FF (*χ*^2^ = 82.34, *p* < 0.001), but not in LC (*χ*^2^ = 2.30, *p* = 0.12) (Figure 5). Overall, in AA two size groups were evident, and high values of betweenness were present only for large nests. In LC, there was not a clear pattern, whereas in FF the majority of high values of betweenness occurred for large nests (Figure 5).

We did not observe any aggressive behavior in the control tests. In the other contests, aggressive behaviors—fighting, biting, and aggressive displays—occurred in 35 (20 inter-site, 15 intra-site) out of 105 tests. In the remaining confrontations, ants showed no aggressive behaviors. Frequency of aggressive behaviors were significantly higher in intra-site than inter-site contests (χ^2^ = 5.09, *p* = 0.024).

## 4. Discussion

Nest mound shape and size differed significantly between the beech forest and both the two other habitats, being on average smaller and flatter in the former than the latter (see Figure 2A,D,G). Nest mound shape can be highly variable, and it is known it is influenced by temperature and humidity variations across habitats, and can be dynamically adapted to cope with exposure to sunlight [37,38]. The observed differences in shape were associated with variations in the building materials. Despite coniferous needles being generally the principal constituent of the nest mounds, *F. paralugubris* behaved opportunistically, also using beech buds when these were widely available and needles were scarcer [21,39]. In our samples, ant nests in both the mixed and the beech forests contained varying amounts of beech buds, with a considerably higher proportion found in the beech forest nests. While beech buds are similar in shape to fir needles, they are hollow and lighter and possess different physical properties. Our results suggest that the mechanical properties of the materials used may be an important determinant of mound shape and size. The material collected from the beech-dominated forest had a narrower angle of repose, suggesting weaker friction forces among the units [29]. In short, increasing amounts of beech buds make growth in height physically difficult, and cause the mounds to be flatter. The angle of repose of the nest material from the mixed forest was similar to that of the fir-dominated forest, suggesting that the small differences in their composition did not have significant structural effect. As the form and size of a mound can affect its functioning in the protection of the underground chambers during harsh winter conditions [2,40], a thorough analysis of the physical properties of the building materials can be an important topic for future investigations on the distribution of RWA in different types of habitats. Finally, we are aware that our experimental approach is an extreme simplification of what really happens in real situations. Building materials are arranged in a specific, overlapping manner so that the nest is protected against the penetration of water, snow, or heat, for example, and all the construction details can contribute to make up the final mound performances.

Despite the evident differences in mound structure and in the interconnection among nests, it appeared that the average volume of nest-networks was similar across sites. Assuming that each network corresponded to a distinct colony, this finding suggests that the average colony size did not vary in the three sites [41], which is quite surprising as the three sites were considerably different, also in terms of potential productivity [42]. Of course, it is possible that not all inter-nest connections could have been observed due to their temporary nature [33,43], and the overall figure could be different. In light of this result, using single nests as an index of colony size could be unreliable for this species [41,44,45]. 

Furthermore, this species displays organizational plasticity in the spatial arrangement of nests according to its habitat. In polydomous ants, the spatial arrangement of nests can be driven by the distribution of resources, e.g., food [46,47] or nesting places [13,22]. The intensity of budding activity can also be variable, for example, when central nests are disturbed [48]. Given the different structure of the three habitats, all of these factors may have had a role in the nest distribution, as well as local environmental features, such as soil depth and texture, slope, and humidity [49]. 

In two of the three habitats analyzed, a positive relation was found between size and centrality—betweenness—of nests. This result is different from the one found in *F. lugubris* and it might suggest a certain central colony-level organization of nest connections in this species [13]. In other words, it appears that larger nests, in the fir-dominated and in beech-dominated forests, have a central placement in the nest networks, suggesting they are the main references for resource distribution, following a typical central place foraging model [50]. However, the imported populations collapsed during the first years after the transplants, and a few surviving nests generated the present populations [25]. Thus, it is possible that some of these nests are simply older, and therefore larger, than the others [51]. 

Finally, low but detectable aggressiveness was observed between ants of different nests. Aggression was more frequent within each population than between different populations. Their low inter-population aggressiveness was consistent with the results from Chapuisat et al. [19], who confronted workers of two populations on the Swiss Alps separated by several hundred meters, or even a few kilometers, a distance similar to that between our sites. On the contrary, the greater number of intra-population aggressions contrasts with the findings by Holzer et al. [52], who did not report any aggressive interaction to occur within the same population, and interactions between non-nestmates reduced to antennation. Our findings suggest that *F. paralugubris* are not completely unicolonial, and that some form of competition among independent colonies still exists. The difference between inter- and intra-population aggressiveness is consistent with the “nasty neighbor” effect [53], a behavior described in several other polydomous but not unicolonial ant species, including the RWA *F. pratensis* [54,55,56]. Unfortunately, the behavior of the population in the Italian Alps, from which the imported population were collected, is not documented, and it is impossible to say whether they are unicolonial like the Swiss populations, or, instead, similar to the ones analyzed in this study. We can be sure that the three studied populations originated from the few nests that survived the introduction [25], and this could have determined a genetic bottleneck, reducing genetic diversity, which in turn may have affected social behavior [57]. A population genetic study and the analysis of cuticular hydrocarbons in the studied populations may shed light on this point.

## 5. Conclusions

In conclusion, we found that spatially close but separated populations of *F. paralugubris* showed considerable plasticity in mound-building and degree of polydomy. The differences observed between the sites were partially driven by habitat-specific features related to the type of forest stand, although it is difficult to draw generalizations as further replicate populations inhabiting similar habitats would be needed. To the best of our knowledge, the FF population is the only one inhabiting a beech forest throughout the introduction range, making replication of the observations impossible. Furthermore, the aggressiveness recorded among sympatric colonies suggests that these imported populations do not behave like the supercolonies described for the unicolonial Alpine populations. The populations imported to Central Italy represent a unique “experiment” of the controlled introduction of RWAs outside their native range of distribution, with the only notable exception being the *F. paralugubris* population introduced to Canada [58], and are therefore a high-value case study. A wider investigation of the fate and ecology of all populations imported into the Apennine range might reveal interesting details on the biology and evolution of introduced ant species.

## Data Availability

Data are available on request.

## Supplementary Materials

The following supporting information can be downloaded at: https://www.mdpi.com/article/10.3390/insects13020198/s1, Table S1: (A) Volumes of nest mounds, (B) size and volumes of networks, and (C) angle of repose for the three sites, and results of all tests performed. In “Nests per network” is reported the range of nests forming networks in that site. Pairwise tests are Tukey post-hoc tests. Sites: AA, Avorniolo Alto; LC, le Cullacce; FF, Fosso Fresciaio. Significance levels of pairwise tests: ns, not significant; *, *p* < 0.05; ** *p* < 0.01; *** *p* < 0.001.
