# Supplementary material for: Intraspecific Relationships and Nest Mound Shape Are Affected by Habitat Features in Introduced Populations of the Red Wood Ant Formica paralugubris
Source: Insects. 2022 Feb 14;13(2):198. doi: 10.3390/insects13020198 (PMC8875456; doi:10.3390/insects13020198)
Supplement: Supplementary file 1 [file insects-13-00198-s001.zip › insects-1542874-supplementary.pdf]

A) Nest mounds

Global test: $F_{2,27} = 13.4$, $p < 0.001$

			Pairwise tests	
	Vol (l)	±SE	AA	LC
AA	901.9	173.7	-	
LC	649.9	140.3	ns	-
FF	253.6	53.2	***	***

B) Networks

Global test (network size): $\chi^2 = 7.19$, $p = 0.027$

Global test (volume): $F_{2,37} = 0.764$, $p = 0.473$

		Pairwise tests			
	Net size	AA	LC	Vol (l)	±SE
AA	1-9	-		3680.2	1647.5
LC	1-7	ns	-	2905.9	967.8
FF	1-35	*	ns	3481.7	2326.1

C) Angle of repose

Global test: $F_{2,27} = 13.4$, $p < 0.001$

			Pairwise tests	
	Ang (rad)	±SE	AA	LC
AA	0.726	0.03	-	
LC	0.656	0.02	ns	-
FF	0.542	0.03	***	**

Table S1. A) Volumes of nest mounds, B) size and volumes of networks, and C) angle of repose for the three sites, and results of all tests performed. In "Nests per network" is reported the range of nests forming networks in that site. Pairwise tests are Tukey post-hoc tests. Sites: AA, Avorniolo Alto; LC, le Cullacce; FF, Fosso Fresciaio. Significance levels of pairwise tests: ns, not significant; *, $p < 0.05$; ** $p < 0.01$; *** $p < 0.001$.
